# Recombinase polymerase amplification - lateral flow dipstick for rapid and visual detection of *Blastocystis spp.*

**DOI:** 10.3389/fcimb.2024.1391943

**Published:** 2024-05-14

**Authors:** Xuefang Mei, Changwei Su, Jiahui Xin, Luwei Jia, Shanrui Zhang, Zhenke Yang, Tian Xiaowei, Zhenchao Zhang, Shuai Wang

**Affiliations:** ^1^ Xinxiang Key Laboratory of Pathogenic Biology, Department of Pathogenic Biology, School of Basic Medical Sciences, Xinxiang Medical University, Xinxiang, Henan, China; ^2^ Department of Laboratory, the Third Affiliated Hospital of Xinxiang Medical University, Xinxiang, Henan, China

**Keywords:** isothermal amplification, *Blastocystis spp.*, lateral flow dipstick, visual detection, rapid detection

## Abstract

*Blastocystis spp.* is a ubiquitous protozoon in the intestinal tract of human and many animals. Microscopic examination is the main method of clinical diagnosis for *Blastocystis spp.*, which is prone to false negative. A simple and rapid diagnosis of *Blastocystis spp.* infection is an important step to prevent and control blastocystosis. Here, a recombinase polymerase amplification-lateral flow dipstick (RPA-LFD) assay was developed for rapid visual detection of *Blastocystis spp.* DNA amplification could be performed within 18 min at 37°C. The minimum DNA detection limit was 1 pg/μL, and there was no cross-reactivity with 12 other non-target pathogens, which was consistent with the sensitivity of conventional PCR (cPCR). Furthermore, 56 fecal samples from the Third Affiliated Hospital of Xinxiang Medical University were tested using RPA and cPCR methods respectively, and the results were completely consistent. The results show that RPA-LFD method has high accuracy and visual results, which provides a new choice for the differential diagnosis and rapid field detection of *Blastocystis spp.*

## Graphical Abstract

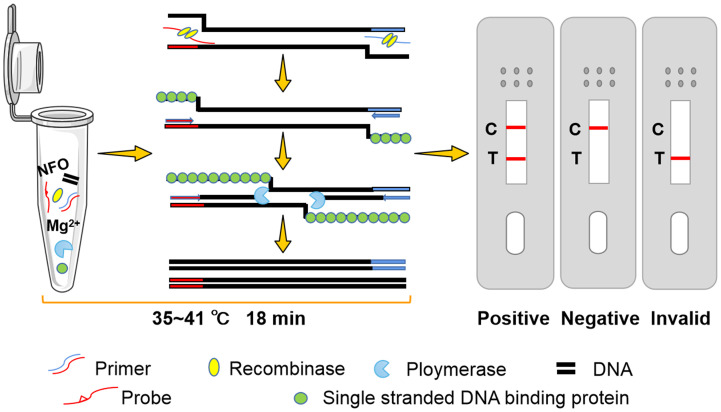

## 1 Introduction

*Blastocystis spp.* is a common intestinal protozoan parasites found in humans and many animals (Mei et al., 2022). The infection rate of *Blastocystis spp.* is about 0.5%~57% in developed countries and 30%~60% in developing countries (Zhang et al., 2019). More than 12 provinces in China have reported *Blastocystis spp.* infection, with a total infection rate of 3.37%. Among them, Fujian Province had the lowest prevalence (0.80%) and Guangdong Province had the highest prevalence (100%) (Deng et al., 2019). Several studies have reported that *Blastocystis spp.* can lead to clinical symptoms such as abdominal pain, diarrhea, vomiting, constipation, and irritable bowel syndrome (IBS) (Légeret et al., 2020; Asghari et al., 2021). In addition, patients with ulcerative colitis develop hematochezia after infection with *Blastocystis spp* (Unalan-Altintop et al., 2022). Rapid and accurate diagnosis of *Blastocystis spp.* infection is an important step in the prevention and control of blastocystosis, which plays an essential role in public health (Liu et al., 2023). However, the clinical manifestations of blastocystosis are non-specific and similar to those caused by other gastrointestinal diseases such as irritable bowel syndrome. Microscopic examination is the main method of clinical diagnosis for *Blastocystis spp* (McHardy et al., 2014)., which is prone to false negative results. Therefore, establishing a rapid, accurate, and convenient detection method for *Blastocystis spp.* has great significance.

Molecular biological diagnosis has great advantages in specificity and sensitivity, and is one of the commonly used diagnostic methods for blastocystosis. Among them, conventional PCR (cPCR) has been widely used in the diagnosis and genotyping of *Blastocystis spp* (Yoshikawa et al., 2004; Stensvold et al., 2006; Domínguez-Márquez et al., 2009; Khademvatan et al., 2018). However, specialized instruments and equipment are required for cPCR, including precise thermal cycling instruments, personnel trained in the technology, and maintaining a cold chain to preserve the heat-unstable reagents. The detection is usually completed in specialized testing centers, which are typically located far away from the high-risk population of infection. In contrast, field detection has certain limitations and has not yet been popularized. Therefore, cPCR detection is usually only performed in the laboratory and rarely used in the initial clinical diagnosis. A more rapid, simple, and accurate detection method is required.

In 2006, recombinase polymerase amplification (RPA) was developed in the UK, providing an isothermal amplification technique for nucleic acids (Piepenburg et al., 2006). The amplification of double-stranded DNA is accomplished through a series of enzyme reactions without thermal or chemical denaturation. Moreover, The results of RPA can be displayed by a lateral flow dipstick (LFD), which is suitable for detection in resource-poor areas or field areas (Wang et al., 2022). RPA-LFD overcomes the need for precision thermal cycling instruments and greatly improves detection efficiency while preserving high sensitivity, thereby allowing rapid visualization of the results (Piepenburg et al., 2006). In recent years, there have been several reports describing the successful application of RPA technology in different parasite detection. including *Giardia duodenalis* (Crannell et al., 2015), *Entamoeba histolytica* (Nair et al., 2015) and *Leishmania Viannia spp* (Saldarriaga et al., 2016).. For *Blastocystis spp.*, only RPA diagnostic methods have been reported (Mei et al., 2023b), it is regrettable that there is no more convenient and visual RPA-LFD diagnostic method at present.

The succinate dehydrogenase subunit A (SDHA) gene is a novel genetic marker utilized in the typing and evaluation of the genetic diversity of *Blastocystis spp* (Higuera et al., 2020).. In this study, an RPA-LFD method targeting the SDHA gene was developed to detect *Blastocystis spp.* rapidly and accurately. This method can be implemented for onsite testing in hospitals or areas with inadequate medical conditions. To our knowledge, this is the first study on the detection of *Blastocystis spp.* using the RPA-LFD technique.

## 2 Materials and methods

### 2.1 DNA samples

The DNA samples used in this study were obtained from Department of Pathogenic Biology, Xinxiang Medical University, including *Blastocystis spp.*, *Dientamoeba fragilis*, *Giardia lamblia*, *Cryptosporidium parvum*, *Ascaris lumbricoides*, *Enterobius vermicularis*, *Trichuris trichiura*, *Clonorchis sinensis*, *Ancylostoma duodenale*, *Lactobacillus taiwanensis*, *Escherichia coli*, *Candida albican*, and *Staphylococcus aureus*. All DNA samples were amplified by cPCR (the primers for cPCR were shown in **Table 1**) and sequenced to confirm the species, and stored at -20°C for use.

**Table 1:** Primers for 12 non-target pathogens.

Species name	Primer/Probe Sequence (5’-3’)
Staphylococcus aureus	F: CACCTGAAACAAAGCATCCTAA R: TATACGCTAAGCCACGTCCAT
Lactobacillus taiwanensis	F: GTCGTCAGCTCGTGTCGTGAGA R: CGCCCGCCGCGCCCCGCGCCCGGCCCGCCG CCCCCGCCCCCCCGGGAACGTATTCACCGCG
Escherichia coli	F: CCGGTTATTGTGAAGGTT R: GTGAATGTTACGGCACTAA
Dientamoeba fragilis	F: CGGACGGCTCATTATAACAGT R: TCCCGTTATTTTCTGAATCACC
Trichuris trichiura Linnaeus	F: ACGT CTGG TTCA GGGT TGTT R: TTAG TTTC TTTT CCTC CGCT
Ascaris lumbricoides	F: ATCGATGAAGAACGCAGC R: TTAGTTTCTTTTCCTCCGCT
Monilia albican	F: CGCCTCTTGATGGTGATGAT R: TCCGGTATCACCTGGCTC
Giardia lambila	F: ATGCCTGCTCGTCGCCCCTTC R: CACTGGCCAAGCTTCTCGCAG
Clonorchis sinensis	F: CGAGGGTCGGCTTATAAAC R: AGCCTCAACCAAAGACAAAG
Ancylostoma duodenale	F: GGATATTGGTACGTTGTATT R: TATAAACCTCAGGATGACCAA
Cryptosporidium parvum	F: AAACCCAACTTTGCGGAAGG R: CCATGCTGGAGTATTCAAGGC
Enterobius vermicularis	F: CTGCGAAAGCATTTGCCAAG R: GCTACCAGCTAAGGCGAGTA

### 2.2 Primer and probe design

The RPA primers and probes were designed to target the SDHA gene (retrieved from the National Center for Biotechnology Information, Gene ID: 24919714) of *Blastocystis spp.* according to the requirements of the RPA assay. Here, three probes and corresponding primers were designed, and the cPCR primers used in this study referred to Scicluna SM et al (Scicluna et al., 2006). (see **Table 2** for details). Sangon Bioengineering Co., Ltd (Sangon, Shanghai, China) was commissioned to synthesize all primers and probes.

**Table 2:** Primers and probe for *Blastocystis spp*.

Assay	Primer/Probe name	Primer/Probe Sequence (5’-3’)
cPCR	BhRDr	F: GAG CTT TTT AAC TGC AAC AAC G
	RD5	R: ATC TGG TTG ATC CTG CCA GT
Primer and probe screening	SDHA-nfo-P1	P: FAM-CAT GAA CGG CGA CGA GTG CGT GGG CGT GAT G-THF-CGC TGT GCC TGG AGG-C3-spacer
	P1-F1	F: TAC TTC ATC GAG TAC CAC GCG CTG GAT CTG
	P1-F2	F: CAC GCT GTA CGG GCG GTC GCT GGC GTA CAA C
	P1-F3	F: TAC CGC ACG GCT GCG GCT GCG GAC CGA ACG
	P1-R1	R: Biotin-TCT GCT TGC AGT GGA AGC GAT GGA TCG ACC
	P1-R2	R: Biotin-TCG CAG AGA AGT AGC AGC GAC CGT AGC CGC
	SDHA-nfo-P2	P: FAM-TCC GCG AGG GCC GCG GCG TGG GCA AGC TGA-THF-GGA CCA CAT CTA CCT-C3-spacer
	P2-F1	F: CCG CGA CGT GGT GTC GCG CGC GAT GAC GAT G
	P2-F2	F: CTT CAT GGA GCG GTA CGC GCC GAC GGC GAA
	P2-F3	F: CTG CCG CGG CGA GGG CGG CGT GCT GCG CAA C
	P2-R1	R: Biotin-TCC GCC AGC AGG TCG GCG GGC AGG TGG TCC
	P2-R2	R: Biotin-TTG TAG TGC ACC GTG GGC AGC ACC GGC ACC
	P2-R3	R: Biotin-TCC ACG CCC GCG AAG ATC ATG GCC GTC TCG
	SDHA-nfo-P3	P: FAM-AGC GCA TGA ACG AGG TGG TGA AGG AGA TCC G-THF-GAC GTG GGC ATC AC-C3-spacer
	P3-F1	F: ACC GCC TGC GCT ACG CGA AGG GCG CCA TCC
	P3-F2	F: CAT GCA GCG CAC CAT GCA GGA CTA CGC GGC
	P3-F3	F: TCC GCA CGG AGG AGA CGC TGC AGG AGG GAT
	P3-R1	R: Biotin-CGA TGA GAT CGG TGT TCC AGA TCA TGG AGC GA
	P3-R2	R: Biotin-ATC ATG GTG CAG TGT GCC TGC TCG AGG AGA
	P3-R3	R: Biotin-GTG GGC TCC ACG CGA CTC CTT GCG CGC CTC
RPA-LFD	P3-F1	F: ACC GCC TGC GCT ACG CGA AGG GCG CCA TCC
	SDHA-nfo-P3	P: FAM-AGC GCA TGA ACG AGG TGG TGA AGG AGA TCC G-THF-GAC GTG GGC ATC AC-C3-spacer
	P3-R3	R: Biotin-GTG GGC TCC ACG CGA CTC CTT GCG CGC CTC

F, forward; R, reverse; LFD, lateral flow dipstick; Biotin, an antigenic marker at 5’-end that binds to a biotin-ligand on the LFD; FAM, carboxyfluorescein; THF, tetrahydrofuran spacer, a site that can be cleaved by NFO to produce a new 3’-hydroxyl group, which converts the probe into a primer; C3 spacer, polymerase extension blocking group.

### 2.3 Primer screening for RPA assay

The forward and reverse primers of each probe were randomly combined, as shown in **Table 3**. The primer screening was performed using commercial basic freeze-dried powder (Amplification future Biotech, Changzhou, China). The process of RPA assay was as follows: 29.4 μL rehydration buffer, 9.1 μL preparation of nuclease-free water, 2 μL forward primer, and 2 μL reverse primer are added into the basic freeze-dried powder and thoroughly mixed. The 5 μL of DNA template was then added to the mixture and 2.5 μL magnesium acetate solution for initiating the reaction was added to the tube cap. The reaction tubes were mixed upside down, centrifuged, and incubated in pre-heated 37°C metal bath for 30 min. Finally, the primer pairs were screened by agarose gel electrophoresis of the amplified products.

**Table 3:** Free combination of forward and reverse primer.

Forward primers	Reverse primers	Amplicon size/bp
P1-F1	P1-R1	112
	P1-R2	157
P1-F2	P1-R1	149
	P1-R2	194
P1-F3	P1-R1	196
	P1-R2	241
P2-F1	P2-R1	117
	P2-R2	210
	P2-R3	165
P2-F2	P2-R1	159
	P2-R2	252
	P2-R3	207
P2-F3	P2-R1	204
	P2-R2	297
	P2-R3	252
P3-F1	P3-R1	201
	P3-R2	250
	P3-R3	287
P3-F2	P3-R1	149
	P3-R2	198
	P3-R3	235
P3-F3	P3-R1	114
	P3-R2	163
	P3-R3	200

### 2.4 Development of the RPA-LFD assay

The PRA-LFD assay was performed as follows: After the reaction Mix (Rehydration buffer, 29.4 μL; forward primer, 2 μL; reverse primer, 2 μL; probe, 0.6 μL; Nuclease-free water, 8.5 μL; 42.5 μL) was prepared, added it to a NFO freeze-dried powder (Amplification future Biotech, Changzhou, China) and mixed well. The primers and probe were shown in **Table 2**. Subsequently, 5μL DNA template and 2.5 μL magnesium acetate solution were added to the suspension, The reaction tubes were incubated in a metal bath at 37°C for 15 min. At the end of the reaction, the LFD (Amplification future Biotech, Changzhou, China) was placed into the reaction solution after 10 times dilution. The band on the LFD determines whether the reaction was positive or negative.

### 2.5 Negative testing of the primers and probes

The primer and probe could easily bind, resulting in false positive in RPA-LFD assay. Therefore, the DNA template of the RPA-LFD assay was replaced with water, and the negative primer and probe was screened through the visualization results of LFD.

### 2.6 Sensitivity analysis

The DNA templates were diluted to 10 pg/μL, 1 pg/μL, 100 fg/μL, and 10 fg/μL, respectively and amplified by RPA-LFD assay. The cPCR assay were performed simultaneously to compare the sensitivity of RPA-LFD assay. The themal cPCR system consists of 12.5 μL of 2×Taq Plus Master Mix, 8.5 μL nuclease-free water, 2 μL DNA, 1 μL forward primer and 1 μL reverse primer. The thermal cycler program settings for cPCR was as follows: predenaturation at 94°C for 5 min; denaturation at 94°C for 45 s, annealing at 59°C for 1 min, extension at 72°C for 1 min, 30 cycles; final extension at 72°C for 10 min.

### 2.7 Determination of specificity

For specificity analysis, 12 non-target pathogens were evaluated by RPA-LFD assay and cRCR assay, including Staphylococcus aureus, Lactobacillus taiwanensis, Escherichia coli, Dientamoeba fragilis, Trichuris trichiura Linnaeus, Ascaris lumbricoides, Monilia albican, Giardia lambila, Clonorchis sinensis, Ancylostoma duodenale, Cryptosporidium parvum, and Enterobius vermicularis.

### 2.8 Optimum reaction time and temperature

The primers and probes screened in the previous experiment were employed for RPA-LFD assay. Different times (3, 6, 9, 12, 15, 18, 21, 24, 27, and 30 min) and different temperatures (25, 30, 35, 37, 39, 41, 43, and 45°C) were set to determine the optimum time and temperature.

### 2.9 Clinical sample testing

A total of 56 clinical fecal samples were collected from the Third Affiliated Hospital of Xinxiang Medical University for RPA-LFD detection. The cPCR assay was also carried out at the same time, and the application effect of RPA-LFD in clinical sample was analyzed by comparing the results of cPCR.

## 3 Results

### 3.1 Primer screening

**Figure 1:**
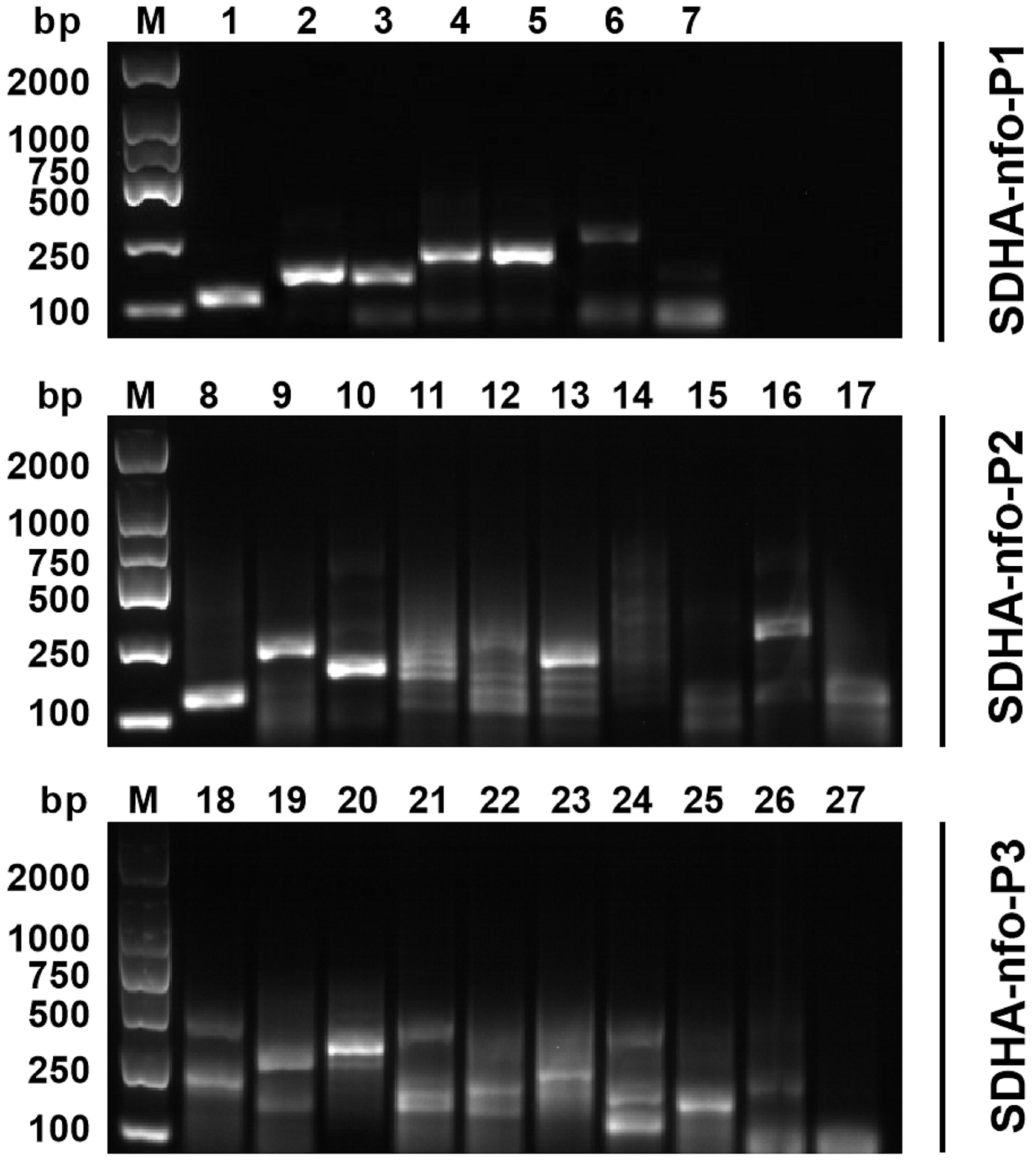
Primer screening for RPA. 1: P1-F1/P1-R1; 2: P1-F1/P1-R2; 3: P1-F2/P1-R1; 4: P1-F2/P1-R2; 5: P1-F3/P1-R1; 6: P1-F3/P1-R2; 7: negative control; 8: P2-F1/P2-R1; 9: P2-F1/P2-R2; 10: P2-F1/P2-R3; 11: P2-F2/P2-R1; 12: P2-F2/P2-R2; 13: P2-F2/P2-R3; 14: P2-F3/P2-R1; 15: P2-F3/P2-R2; 16: P2-F3/P2-R3; 17: negative control; 18: P3-F1/P3-R1; 19: P3-F1/P3-R2; 20: P3-F1/P3-R3; 21: P3-F2/P3-R1; 22: P3-F2/P3-R2; 23: P3-F1/P3-R3; 24: P3-F3/P3-R1; 25: P3-F3/P3-R2; 26: P3-F3/P3-R3; 27: negative control; M, DNA marker.

Five pairs of primers were selected according to the results of agarose gel electrophoresis, including P1-F1/P1-R2, P1-F3/P1-R1, P2-F1/P2-R1, P2-F1/P2-R3, and P3-F1/P3-R3 (**Figure 1**).

### 3.2 Negative testing of primers and probes

**Figure 2:**
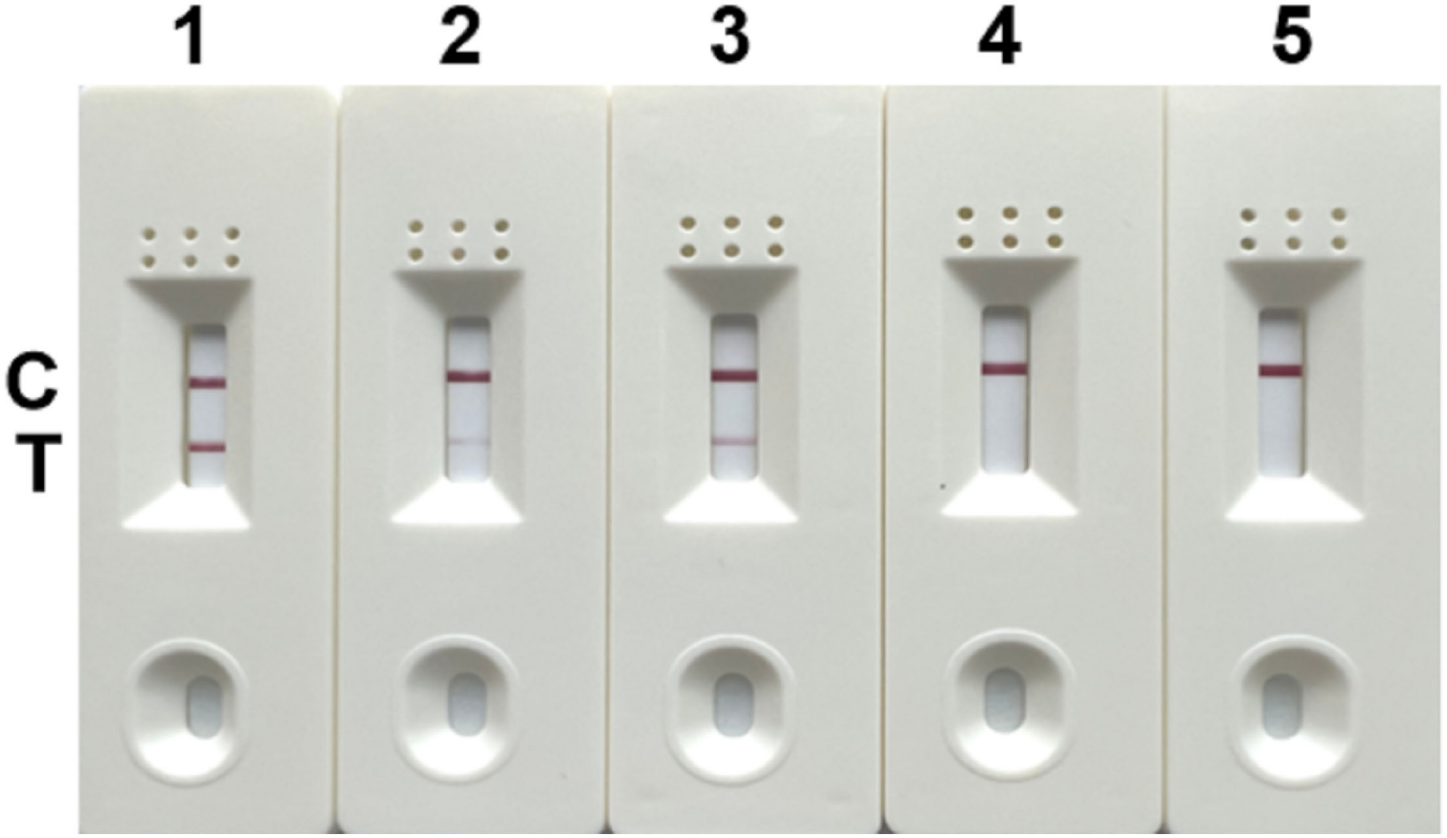
Negative testing of the primers and probes. 1: SDHA-nfo-P1/P1-F1/P1-R2; 2: SDHA-nfo-P1/P1-F3/P1-R1; 3: SDHA-nfo-P2/P2-F1/P2-R1; 4: SDHA-nfo-P2/P2-F1/P2-R3; 5: SDHA-nfo-P3/P3-F1/P3-R3; C, control line; T, test line.

Negative testing was performed for the 5 pairs of primers and probes. The results showed false positives with the combinations of SDHA-nfo-P1/P1-F1/P1-R2, SDHA-nfo-P1/P1-F3/P1-R1, and SDHA-nfo-P2/P2-F1/P2-R1. In contrast, the combinations of SDHA-nfo-P2/P2-F1/P2-R3, SDHA-nfo-P3/P3-F1/P3-R3 were negative which could be further screened for sensitivity (**Figure 2**).

### 3.3 Sensitivity analysis

**Figure 3:**
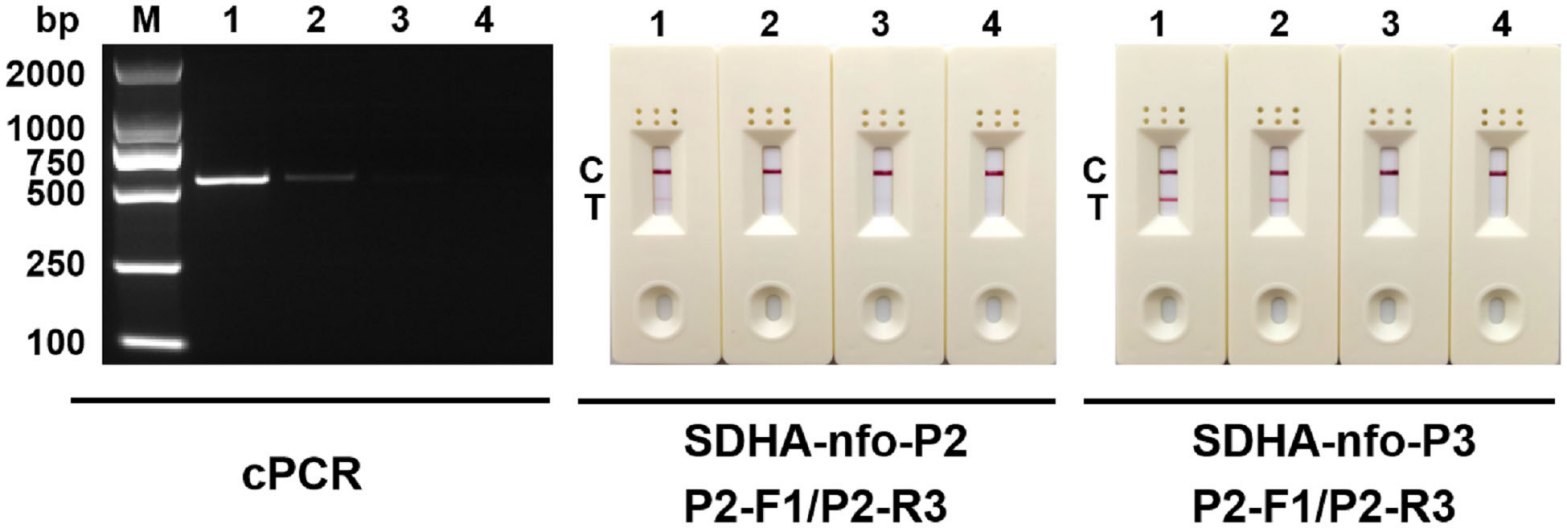
Sensitivity analysis. Different concentrations of DNA were used for cPCR assay and RPA-LFD assay. 1: 10 pg/μL; 2: 1 pg/μL; 3: 100 fg/μL; 4: 10 fg/μL; M, DNA marker; C, control line; T, test line.

The results of the sensitivity test are displayed in **Figure 3**. Among the 2 pairs of primers and probes, SDHA-nfo-P3/P3-F1/P3-R3 showed the highest sensitivity. The lowest concentration of DNA that could be detected was 1 pg/μL when the RPA-LFD assay was performed using SDHA-nfo-P3/P3-F1/P3-R3, which was consistent with cPCR. The findings demonstrated that the RPA-LFD had high sensitivity, and the samples showed positive results when the concentration of *Blastocystis spp.* was above 1 pg/μL.

### 3.4 Determination of specificity

**Figure 4:**
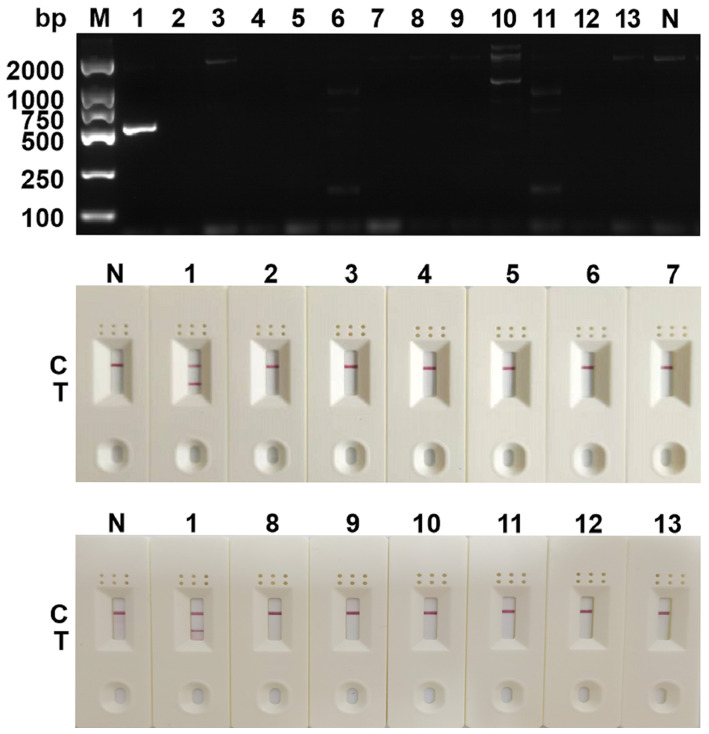
Determination of specificity. The specificity analysis of cPCR and RPA-LFD assay. 1: *Blastocystis spp.*; 2: *Staphylococcus aureus*; 3: *Lactobacillus taiwanensis*; 4: *Escherichia coli*; 5: *Dientamoeba fragilis*; 6: *Trichuris trichiura Linnaeus*; 7: *Ascaris lumbricoides*; 8: *Monilia albican*; 9: *Giardia lambila*; 10: *Clonorchis sinensis*; 11: *Ancylostoma duodenale*; 12: *Cryptosporidium parvum*; 13: *Enterobius vermicularis*; M, DNA marker; N, negative control; C, control line; T, test line.

The previously screened SDHA-nfo-P3/P3-F1/P3-R3 were further verified by specificity test. As shown in **Figure 4**, no false positives or false negatives were found in the RPA-LFD and cPCR, suggesting that the RPA-LFD method has high specificity.

### 3.5 Optimum reaction time and temperature

**Figure 5:**
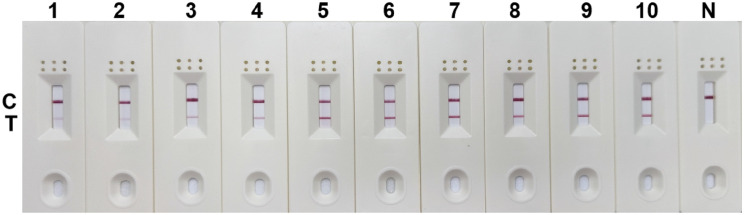
Optimum reaction time for RPA-LFD assay. 1: 3 min; 2: 6 min; 3: 9 min; 4: 15 min; 5: 18 min; 6: 21 min; 7: 24 min; 8: 27min; 9: 30 min; N, negative control; C, control line; T, test line.

**Figure 6:**
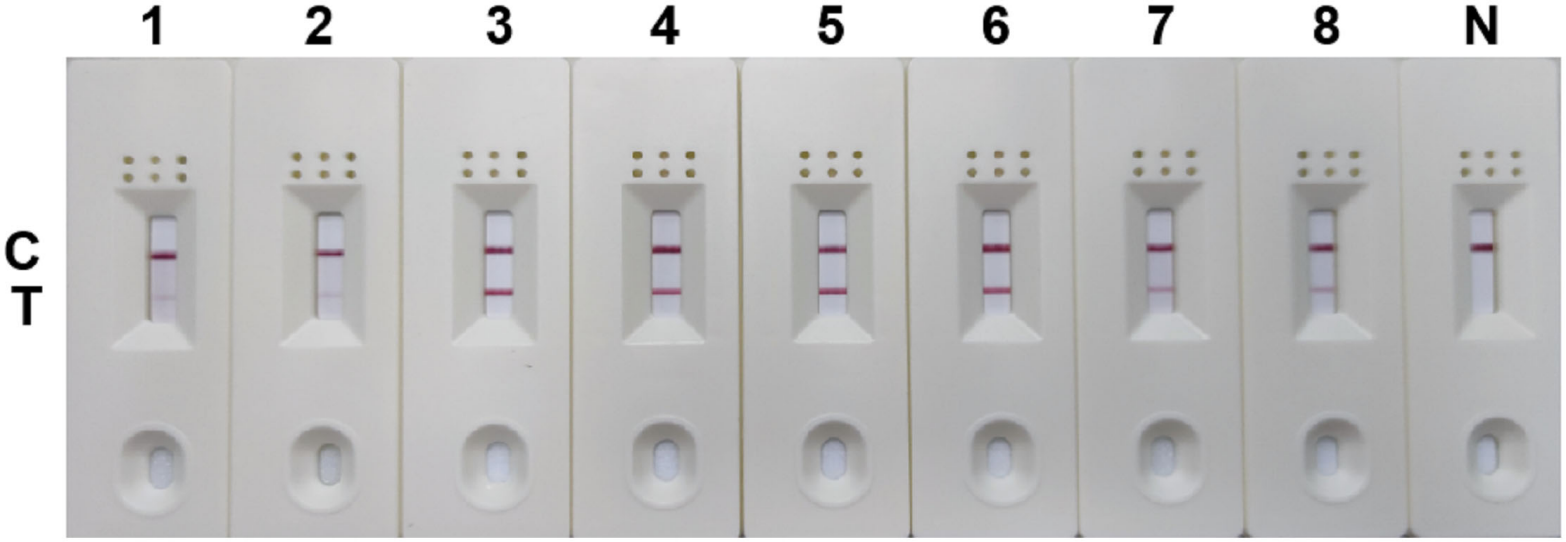
Optimum reaction temperature for RPA-LFD assay. 1: 25°C; 2: 30°C; 3: 35°C; 4: 37°C; 5: 39°C; 6: 41°C; 7: 43°C; 8: 45°C; N, negative control; C, control line; T, test line.

The RPA-LFD assay was conducted with a combination of primer and probe (SDHA-nfo-P3/P3-F1/P3-R3) at different reaction times (3, 6, 9, 12, 15, 18, 21, 24, 27, and 30 min) and different reaction temperatures (25, 30, 35, 37, 39, 41, 43, and 45°C). The test line of the RPA reaction could be observed in the LFD as from 3 min. The detection line was clearest when the RPA reaction lasted for 18 min (**Figure 5**), and no significant change was observed when the reaction time was extended. The reaction was successfully completed in the temperature range of 25~45°C, and the bands are similar at 35~41°C (**Figure 6**). 37°C was selected as the final reaction temperature of this method for clinical sample detection.

### 3.6 Application effect of clinical sample detection

**Figure 7:**
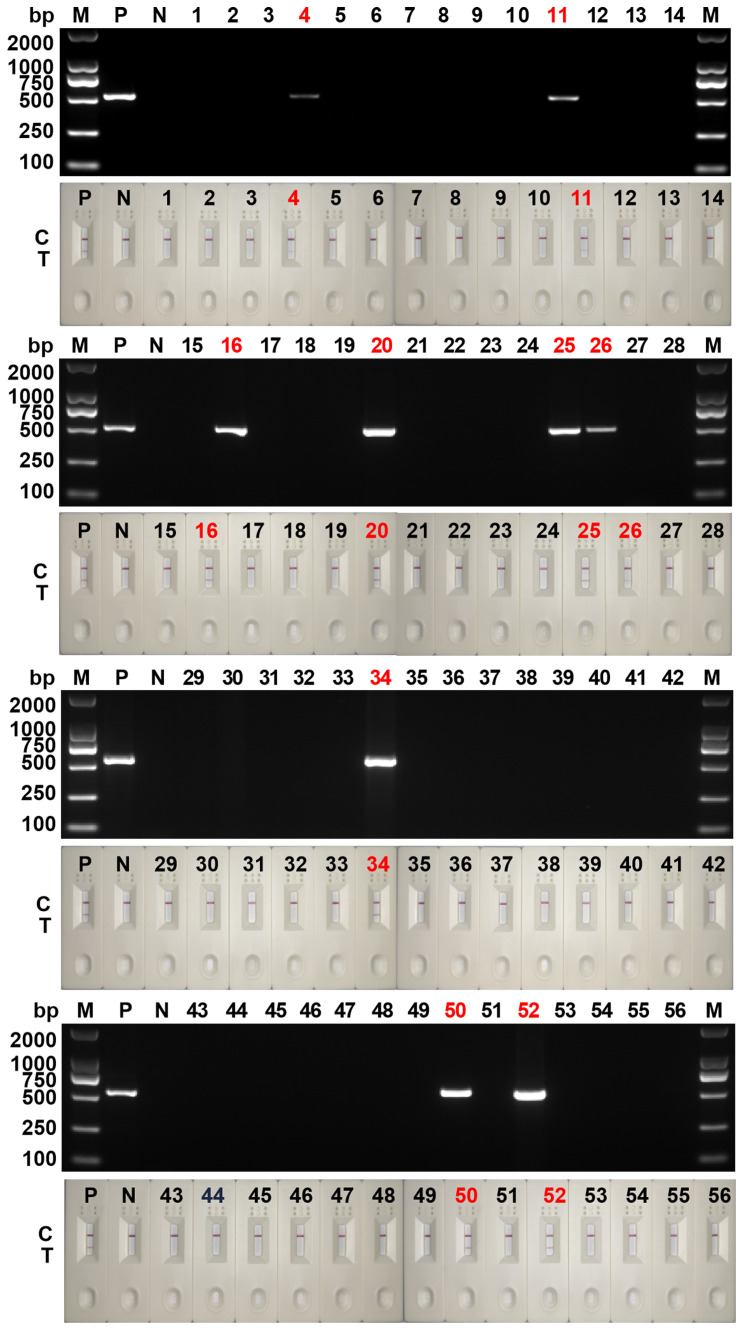
Clinical sample testing for cPCR assay and RPA-LFD assay. 1~56: the numbers of 56 clinical samples; M, DNA marker; P, positive control; N, negative control; C, control line; T, test line.

The detection results from 56 clinical samples showed that 9 fecal samples were positive for *Blastocystis spp.* and 47 were negative (**Figure 7**). The results of RPA-LFD were consistent with those of cPCR. Further sequencing results showed that 9 positive samples contained 3 subtypes, namely ST3 (6/9, 66.7%), ST4 (1/9, 11.1%) and ST7 (2/9, 22.2%).

## 4 Discussion

A variety of intestinal pathogens, including viruses, bacteria and parasitic protozoa, could cause diarrhea, which is the second leading cause of death among children (Kotloff et al., 2013; Caner et al., 2020; dos Santos David et al., 2022; Kotloff, 2022). Among them, the cause of chronic diarrhea is increasingly thought to be caused by parasitic protozoa infection (Lima and Guerrant, 1992), such as *Blastocystis spp.*, *Giardia spp.*, *Entamoeba spp.* Among them, *Blastocystis spp.* is the most widely distributed protozoa (Kaewjai et al., 2023). Similar clinical symptoms and different treatment methods highlight the importance of establishing a rapid and accurate diagnostic method for *Blastocystis spp.*

In this study, a simple, rapid, sensitive, and accurate diagnostic method for *Blastocystis spp.* was achieved by RPA-LFD assay. This method resolved the low sensitivity limitations of microscopic observation and the requirement for expensive equipment for cPCR in remote areas. Furthermore, this method has high sensitivity, and the samples showed positive results when the DNA concentration of *Blastocystis spp.* was greater than 1 pg/μL. The species with the same colonization site or close relationship with the target pathogen were prone to false positives in molecular diagnosis. The RPA-LFD method established in this study was proved to have no cross-reaction with these non-target pathogens, indicating that the method has good specificity.

In contrast to PCR, which can be heated to avoid non-specific amplification caused by binding between primers, nucleic acid amplification achieved by RPA under constant temperature easily generates primer dimers. The formation of primer dimers not only affects the amplification efficiency but also may affect the test results in case of low or no template concentration (Özay and McCalla, 2021). To overcome this limitation, several RPA primers and probes were designed with the SDHA gene as the target sequence. Five pairs of primers with higher amplification efficiency were screened by RPA assay. Subsequently, two pairs of primers and probes passed negative testing. In the further sensitivity test, SDHA-nfo-P3/P3-F1/P3-R3 showed the highest sensitivity, which then passed the specificity test. Therefore, SDHA-nfo-P3/P3-F1/P3-R3 was finally determined to be the best combination primer and probe.

The RPA-LFD assay was effectively carried out at 25~45°C, indicating that the RPA-LFD assay had lower requirements for temperature conditions and a wider detection range, which was more convenient for the detection of *Blastocystis spp* (Abd El Wahed et al., 2015).. In addition, existing RPA methods require agarose gel electrophoresis to read the results (Mei et al., 2023b). In contrast, the RPA-LFD method established in this study can make a diagnosis faster, as the LFD can observe the T-line within 3 min. The accuracy of the method was further confirmed by the detection of clinical samples. However, the distribution of different subtypes of *Blastocystis spp.* is related to many factors such as geographical location and season. The clinical samples used in this study were from Xinxiang City, China. It was reported that there were 8 subtypes (ST1-ST7, ST12) in the Chinese, and only ST1, ST3, ST4, ST6 and ST7 were reported in Xinxiang City (Deng et al., 2019; Zhang et al., 2019; Mei et al., 2023a). Although only ST3, ST4 and ST7 were included in the 56 clinical samples tested here, the results of RPA-LFD were completely consistent with those of cPCR, indicating that this method has good clinical application potential. In future studies, clinical samples containing more subtypes of *Blastocystis spp.* can be tested to further ensure the accuracy and stability of the method.

## 5 Conclusions

In summary, a rapid detection method for *Blastocystis spp.* was developed using RPA technology, with the detection results being visualized by LFD. This method overcame the requirements of cPCR for thermal cycling instruments and agarose gel electrophoresis instruments. Moreover, amplification was effectively completed in the temperature range of 25~45°C. The detection time was also greatly shortened, and the results were observed by LFD after 3 min of RPA reaction. The lowest detection limit of DNA was 1 pg/μL, which was consistent with the cPCR. In addition, this method showed good specificity. This study provided a simple, rapid, and accurate method for the diagnosis of *Blastocystis spp*.

## Data availability statement

The original contributions presented in the study are included in the article/supplementary material. Further inquiries can be directed to the corresponding author.

## Ethics statement

The studies involving humans were approved by the Ethical Commission of the Xinxiang Medical University. The studies were conducted in accordance with the local legislation and institutional requirements. The participants provided their written informed consent to participate in this study. The manuscript presents research on animals that do not require ethical approval for their study. Written informed consent was obtained from the individual(s) for the publication of any potentially identifiable images or data included in this article.

## Author contributions

XM: Funding acquisition, Methodology, Writing – original draft, Writing – review & editing. CS: Data curation, Formal analysis, Investigation, Methodology, Writing – original draft. JX: Formal analysis, Investigation, Writing – original draft. LJ: Data curation, Investigation, Writing – original draft. SZ: Data curation, Investigation, Writing – original draft. ZY: Formal analysis, Writing – original draft. TX: Conceptualization, Formal analysis, Writing – original draft. ZZ: Conceptualization, Writing – original draft. SW: Conceptualization, Funding acquisition, Resources, Writing – review & editing.
